# Mathematical modeling and thermodynamics of Prandtl–Eyring fluid with radiation effect: a numerical approach

**DOI:** 10.1038/s41598-021-01463-4

**Published:** 2021-11-12

**Authors:** Zakir Ullah, Ikram Ullah, Gul Zaman, Hamda Khan, Taseer Muhammad

**Affiliations:** 1 Department of Mathematics, University of Malakand, Chakdara, Dir(L), Khyber Pakhtunkhwa 18800 Pakistan; 2 Department of Sciences and Humanities, National University of Computer and Emerging Sciences, Peshawar, KP 25000 Pakistan; 3 Department of Sciences and Humanities, National University of Computer and Emerging Sciences, Islamabad, Pakistan; 4 Department of Mathematics, College of Sciences, King Khalid University, Abha, 61413 Saudi Arabia

**Keywords:** Fluid dynamics, Statistical physics, thermodynamics and nonlinear dynamics, Applied mathematics

## Abstract

Main concern of current research is to develop a novel mathematical model for stagnation-point flow of magnetohydrodynamic (MHD) Prandtl–Eyring fluid over a stretchable cylinder. The thermal radiation and convective boundary condition are also incorporated. The modeled partial differential equations (PDEs) with associative boundary conditions are deduced into coupled non-linear ordinary differential equations (ODEs) by utilizing proper similarity transformations. The deduced dimensionless set of ODEs are solved numerically via shooting method. Behavior of controlling parameters on the fluid velocity, temperature fields as well as skin friction and Nusselt number are highlighted through graphs. Outcome declared that dimensionless fluid temperature boosts up for both the radiation parameter and Biot number. It is also revealed that the magnitude of both heat transfer rate and skin friction enhance for higher estimation of curvature parameter. Furthermore, comparative analysis between present and previous reports are provided for some specific cases to verify the obtained results.

## Introduction

In fluid dynamics, the phenomenon of stagnation-point flow has got considerable attention of various researchers in the recent past due to its significant applications in natural and industrial phenomena. The former includes a flow of fluid over the tips of various objects, e.g., ships, submarines, aircrafts, rockets etc^1^. In biology, a blood-flow in the blood vessel at the branch/ sub-branch separates into two or more directions and corresponds to the stagnation-point flow^2^. Hiemenz^3^ in 1911, first proposed an exact solution for the stagnation-point flow in a static-rigid surface. In this study, Hiemenz utilized appropriate transformation to transform the steady two dimensional (2D) Navier-Stokes equations into non-dimensional highly ODEs. After the remarkable work of Hiemenz^3^, many investigators considered the stagnation-point flow phenomena by means of different physical features^4–7^. Recently, Vaidya et al.^8^ examined the steady 2D oblique stagnation-point flow on a stretching plate. They have solved analytically dimensionless highly non-linear ODEs using the Optimal Homotopy Analysis Method (OHAM). Further, it has been shown there^8^ that axial fluid velocity declines with a rise in the viscosity while the dual effect of viscosity is found on the transverse fluid velocity. Meanwhile, Hayat et al.^9^ discussed the steady 2D stagnation-point flow with both heat generation and thermal radiation. They noticed in^9^ that variations in the radiation variable and Biot number improve the dimensionless fluid temperature. Further, Aly and Pop^10^ have obtained unique and dual solutions for a steady 2D stagnation-point flow associated with dynamic hybrid nanofluid. They showed that dual and unique solutions exist for a certain estimations of magnetic parameter and revealed that the behavior of hybrid nanofluid velocity field and temperature are different along the three regions of stability. Additionally, Wain et al.^11^ comprehended the analysis for incompressible stagnation-point flow in a shrinking/stretching plate, admitting growth of skin friction and heat transfer due to the melting parameter.

Non-Newtonian fluids flow phenomena plays a pivotal role in numerous natural, industrial, geophysical and engineering processes. Some common examples of these fluids are drilling mud, lubricating oils, liquid crystals, paints, silly putty, polymeric liquids, biological fluids and many others. The properties of such fluids are hard to define as a single constitutive equation but many attempts have been made by the investigators to characterize the rheological characteristics of fluids containing non-Newtonian fluid behavior. Non-Newtonian fluid models are evidently more complex and have a highly nonlinear behavior. Various investigators presented different fluid models^12–27^ to describe the complex nature of non-Newtonian fluids phenomena. Prandtl–Eyring model is a particular type of non-Newtonian fluid which indicates that shear stress is proportional to the sine hyperbolic function of strain rate to the fluid. Recently, Khan et al.^28^ proposed the combined impacts of Brownian and thermophoresis diffusion on 2D Prandtl–Eyring nanofluid with entropy generation through a heated stretchable plate. They revealed that for greater estimations of Brinkman number and material parameter, the entropy generation rate rises. Further, the influences of heat source and thermophoresis on steady incompressible MHD flow of Prandtl–Eyring nanofluid in a symmetric channel was analyzed by Akram et al.^29^. They analyzed that Brownian and thermophoresis parameters have opposite behavior on both the temperature gradient and heart transfer rate. Meanwhile, Uddin et al.^30^ examined numerically the impact of activation energy on dynamical 2D MHD Prandtl–Eyring nonofluid due to the Joule heating effect. Additionally, Rehman et al.^31^ studied scaling group transformation method for steady incompressible Prandtl–Eyring fluid through a 2D semi-infinite stretching sheet. With the help of scaling transformation they obtained new similarity transformations for the analysis of Prandtl–Eyring fluid flow. Abdelsalam et al.^32^ used the Eyring-Powell fluid model as the base fluid to investigate the behavior of a microorganism swimming through a cervical canal. Moreover, Shankar and Naduvinamani^33^ carried out the numerical solution for magnetized squeezed unsteady 2D Prandtl–Eyring fluid flow through a horizontal sensor sheet. From their investigation it has been noticed that fluid velocity boosts with magnetic parameter while the fluid temperature diminishes in the flow region with magnetic parameter.

The influence of thermal radiation plays an essential role in space technology and in processes with high temperatures. The study of heat transfer characteristics on a stretched sheet with radiation was studied by a number of researchers. Smith^34^ was the first researcher who presented the aspect of thermal radiation on steady 2D flow. Later on, the influence of thermal radiation on fluid temperature and heat transfer in an emitting/absorbing medium flowing on a wedge was explored by Viskanta and Grosh^35^. Recently, Raza et al.^36^ numerically elaborated the impacts of MHD and thermal radiation on unsteady 2D molybdenum disulfide nanoparticle through a porous channel. They revealed that the heat transfer rises by enhancing the solid volume fraction for various shapes of nanofluids. Gireesha et al.^37^ analyzed the preparation process of hybrid nanomaterials on a porous longitudinal fin with thermal radiation. Wakif^38^ scrutinized the impact of incompressible MHD flow of Casson fluid on a horizontal stretched plate with thermal radiation and they show that with radiation parameter the nanofluid temperature increases. Additionally, the characteristics of heat transfer and MHD nanoparticle on a stretching plate with thermal radiation and Joule heating impacts was scrutinized by Dogonchi and Ganji^39^. They observed that with an increase in the volume of nanofluid turn out a linear rise in the Nusselt number, whereas, this number shows inverse behavior with thermal radiation. Khan and Alzahrani^40^ proposed the combined effects of thermal radiation and viscous dissipation on 2D nanofluid with entropy generation through a stretched surface. Raza et al.^41^ studied the thermal radiation impacts on the convective flow of a non-Newtonian fluid through a curved surface. Moreover, Ullah et al.^42^ numerically studied the flow pattern followed by hybrid nanoliquids (AA7075, AA7072) using an infinite disk in the presence of thermal radiation. Furthermore, the authors suggested that Nusselt number shows direct behavior with thermal slip and radiation parameters where reverse effect was noticed for large Eckert number.

In view of aforementioned literature survey, it is concluded that Prandtl–Eyring fluid in the cylindrical geometry is not addressed yet. Therefore our intention here is to develop a novel mathematical modeling for incompressible MHD^43,44^ Prandtl–Eyring fluid flow near the stagnation-point induced by stretching cylinder. Energy expression is characterized with thermal radiation. Suitable transformations are utilized to convert the set of non-linear PDEs into a system of highly non-linear ODEs. The reduced dimensionless system is then solved by Shooting method. The influence of various controlling parameters and dimensionless numbers, like curvature, magnetic, radiation and fluid parameters, Prandtl and Biot numbers on the fluid velocity, temperature as well as skin friction and heat transfer are reported via graphs and investigated. The present results of skin friction and heat transfer rate are compared with the previous published work in the limiting cases which are found to be satisfactory.

## Mathematical modeling

**Figure 1:**
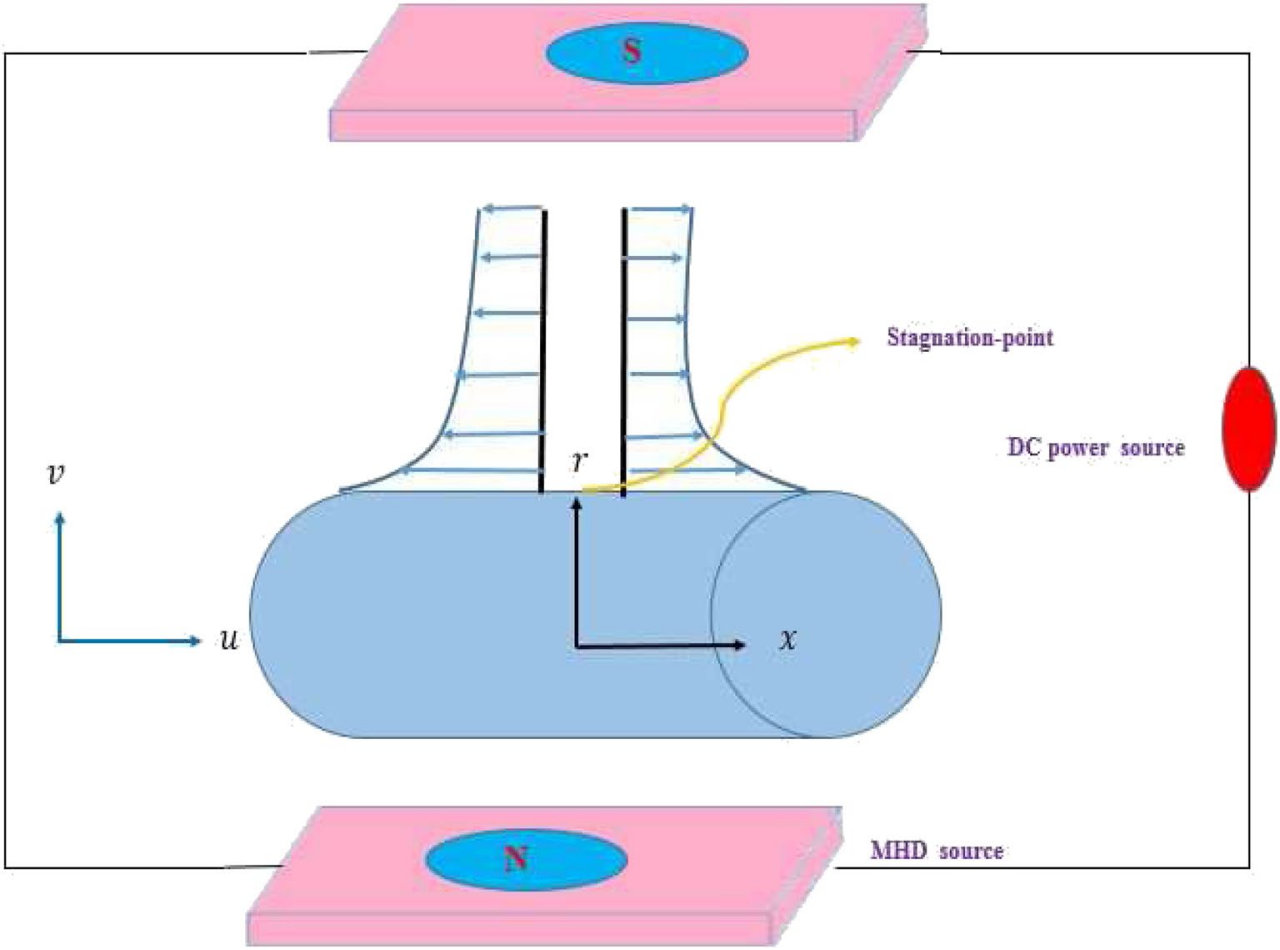
Flow configuration.

We consider steady, axisymmetric and 2D MHD stagnation-point flow of incompressible Prandtl–Eyring fluid model by a stretching cylinder. Radiation is considered in the heat expression. Further, let the cylinder is being Stretchable in the $x$-axis with linear velocity $u=\frac{U_0 x}{l}$. Let the respective (*x*, *r*)-coordinates are presumed in cylinder and normal to it (see Fig. 1). Moreover, heat transportation is performed under the convective surface condition. The constitutive equation for the Prandtl–Eyring fluid model^45^ is given as

1 $$\begin{aligned} T=-pI+\mu S. \end{aligned}$$

In Eq. (1), *T*, *p*, *I* and $\mu$ are fluid Cauchy stress tensor, fluid pressure, identity tensor, and dynamic viscosity respectively. Where *S* strands for extra stress tensor of Prandtl–Eyring fluid model and given as follows^45^:

2 $$\begin{aligned} S = \left[ \frac{a_1 ~arc~\sinh\left( \frac{1}{c_1}\sqrt{\frac{1}{2}tr(A_1 ^2)}\right) }{\sqrt{\frac{1}{2}tr(A_1 ^2)}}\right] A_1. \end{aligned}$$

In Eq. (2), $a_1$ and $c_1$ denotes the material parameters of fluid and $A_1 = \nabla V + (\nabla V)^T$ is the first Rivlin-Ericksen tensor. The first Rivlin-Ericksen tensor $A_1$ for present study in cylindrical coordinates is expressed as

3 $$\begin{aligned} A_1 = \begin{bmatrix} 2\frac{\partial v}{\partial r} &{} 0 &{} \frac{\partial u}{\partial x} +\frac{\partial u}{\partial r} \\ 0 &{} 2\frac{v}{r} &{} 0 \\ \frac{\partial u}{\partial r} +\frac{\partial u}{\partial x} &{} 0 &{} 2\frac{\partial v}{\partial x} \end{bmatrix}. \end{aligned}$$

The required component of the present model is given by

4 $$\begin{aligned} \tau _{rx}=\left[ \frac{a_1}{\rho }~arc~ \sinh\left( \frac{1}{c_1}\frac{\partial u}{\partial r}\right) \right] , \end{aligned}$$

here $\sinh^{-1}$ is presumed upto second-order estimation and is expressed by

5 $$\begin{aligned} \sinh^{-1}\left( \frac{1}{c_1}\frac{\partial u}{\partial r}\right) =\frac{1}{c_1}\frac{\partial u}{\partial r} -\frac{1}{6}\left( \frac{1}{c_1}\frac{\partial u}{\partial r}\right) ^3. \end{aligned}$$

Under the above assumption, the flow governing expressions are^46–48^

6 $$\begin{aligned}\frac{\partial (ru)}{\partial x}+\frac{\partial (rv)}{\partial r}=0, \end{aligned}$$

7 $$\begin{aligned}\\ \\\quad u\frac{\partial u}{\partial x}+v\frac{\partial u}{\partial r}=U_e \frac{dU_e}{dx}+\frac{a_1}{\rho c_1}\left[ \frac{1}{r}\frac{\partial u}{\partial r}+\frac{\partial ^2 u}{\partial r^2}\right] -\frac{a_1}{2\rho c_1 ^3}\left( \frac{\partial u}{\partial r}\right) ^2\left( \frac{\partial ^2 u}{\partial r^2}\right) - \frac{a_1}{6\rho c_1 ^3 r}\left( \frac{\partial u}{\partial r}\right) ^3 +\frac{\sigma B_0 ^2}{\rho }(U_e-u), \end{aligned}$$

8 $$\begin{aligned}u\frac{\partial T}{\partial x}+v\frac{\partial T}{\partial r}=\alpha \left[ \frac{\partial ^2 T}{\partial r^2}+\frac{1}{r}\frac{\partial T}{\partial r}\right] +\frac{1}{\rho c_p}\frac{16\sigma ^* T_\infty ^3}{3k^*}\left[ \frac{\partial ^2 T}{\partial r^2}+\frac{1}{r}\frac{\partial T}{\partial r}\right] , \end{aligned}$$

along with associated boundary conditions^47,48^

9 $$\begin{aligned}u=U(x)=\frac{U_0 x}{l},~~ v= 0, ~~ -k\frac{\partial T}{\partial r}=h_f(T_f-T\infty ) \ \ \text {at} ~~~~ r=R, \end{aligned}$$

10 $$\begin{aligned}u\rightarrow U_e (x)=\frac{U_\infty x}{l}, ~~ \ T\rightarrow T_\infty ~~~~ \text {as} ~~~~ r\rightarrow \infty . \end{aligned}$$

In which *u* and *v* represents the respective velocity in the $x$- and $r$-directions , *T*, $T_w$ and $T_\infty$, indicates fluid, boundary and free stream temperatures respectively, the symbols $\nu$, $B_0$ and $\sigma$ denotes kinematic viscosity, strength of magnetic field and liquid electrical conductivity respectively. The thermal diffusivity, coefficient of mean absorption, fluid density, specific heat and Stefan-Boltzmann constant are denoted respectively by the symbols $\alpha$, $k^*$, $\rho$, $c_p$ and $\sigma ^*$.

Now, considering the following similarity variables

11 $$\begin{aligned} \eta =\frac{r^2-R^2}{2R}\sqrt{\frac{U_0}{l\nu }}, ~~~~ \psi =\sqrt{\frac{U_0\nu }{l}}Rxf(\eta ), ~~~ \theta (\eta )=\frac{T-T_\infty }{T_f-T_\infty }, \end{aligned}$$

where

12 $$\begin{aligned} u=\frac{1}{r}\frac{\partial \psi }{\partial r} ~~~\text {and} ~~~~ v=-\frac{1}{r}\frac{\partial \psi }{\partial x}. \end{aligned}$$

Using Eq. (11) along with Eq. (12) in Eqs. (6)–(10), gives

13 $$\begin{aligned}A\left[ 2Kf{''}+(2K\eta + 1)f{'''}\right] -A\beta \left[ \frac{4}{3}K(1+2K\eta )(f{''})^3+(1+2K\eta )^2(f{'''})(f{''})^2\right] \nonumber \\&\quad +f{''} f - (f')^2 + M^2(B-f') + B^2=0 , \end{aligned}$$

14 $$\begin{aligned}\left( 1+\frac{4}{3}R\right) \left[ (1+2K\eta )\theta {''}+2K\theta {'}\right] +Prf\theta {'}=0, \end{aligned}$$

15 $$\begin{aligned}f(0)=0, ~~ \ f'(0)= 1, ~~ \theta {'}(0)=-Bi\left( 1-\theta (0)\right) , \ \end{aligned}$$

16 $$\begin{aligned}f'(\infty )= B, ~~ \theta (\infty )=0. \end{aligned}$$

In the above expressions $A= \frac{a_1}{\mu c_1}$ and $\beta = \frac{U_0 ^3 x^2}{2c_1 l^3 \nu }$ denoted fluid parameters, $K=\frac{1}{R}\sqrt{\frac{l\nu }{U_0}}$ indicates the curvature parameter, $M=\sqrt{\frac{l\sigma B_0^2}{U_0\rho }}$ means a magnetic field parameter, $B=\frac{U_\infty }{U_0}$ is the ratio of velocities, $Pr=\frac{\nu }{\alpha }$ denotes the Prandtl number, $R=\frac{4\sigma ^* T_\infty ^3}{k*k}$ denotes radiation parameter and $Bi=\sqrt{\frac{l\nu }{U_0}}\frac{h_f}{k}$ is the Biot number.

Finally, the mathematical expressions for the important aspects i.e., skin friction coefficient $(C_{f_{x}})$ and the Nusselt number $Nu_x$ are given by

17 $$\begin{aligned} C_{f_{x}} = \frac{\tau _w}{\frac{\rho U_0^2 x^2}{2l^2}}, ~~ Nu_x = \frac{xq_w}{k(T_f -T_\infty )} . \end{aligned}$$

In Eq. (17), the wall shear stress and heat flux respectively are

18 $$\begin{aligned} \tau _w = a_1\left[ \frac{1}{c_1}\frac{\partial u}{\partial r}-\frac{1}{6c_1 ^3}\left( \frac{\partial u}{\partial r}\right) ^3\right] _{r=R}~~ q_w = \left( k+\frac{16\sigma ^*T_\infty ^3}{3k^*}\right) \left( \frac{\partial T}{\partial r}\right) _{r=R}. \end{aligned}$$

Inserting Eq. (11) along with Eq. (12) into Eq. (17) we obtain

19 $$\begin{aligned} \frac{Re^{\frac{1}{2}}C_{f_x}}{2} = Af{''}(0)-\frac{1}{3}A\beta f{''}^3(0), ~~~ Re^{-\frac{1}{2}}Nu_x = -\left( 1+\frac{4}{3}R\right) \theta ^{'} (0), \end{aligned}$$

where represents the local Reynolds number and can be expressed as $Re = \frac{U_0 x^2}{l\nu }$.

## Numerical scheme

The obtained dimensionless system of ODEs and validation analysis together with the appropriate conditions cannot be simulated directly or analytically due to highly non-linear nature . Therefore, these non-linear ODEs are solved numerically by implementing Shooting iterative technique via Mathematica software. Here, in this numerical procedure first higher order ODEs in Eqs. (13) and (14) are altered into a set of first order ODEs. In this numerical procedure, it is also very significant to assume an appropriate finite value for $\eta \rightarrow \infty$. Furthermore, we also choose suitable initial guesses of $f''(0)$ and $\theta '(0)$ and obtain the solution by adopting Runge-Kutta Fehlberg fifth order technique as an initial value problem which has truncation error of order 5. The accuracy of the current results has been verified and are given in Tables 1, 2, 3 by comparing with the existing solutions of^49–54^ for some particular cases, where it is revealed that the current results and their solutions are approximately identical.

**Table 1:** Comparative values of skin friction $Re^{\frac{1}{2}}C_{f_x}$ against variations in *M* when $A=1, K = \beta = B = Pr = R = Bi = 0.0$.

*M*	Ref.^49^	Ref.^50^	HAM results	Present results
0	$-1$	$-1$	$-1$	$-1$
0.5	$-1.11803$	$-1.1180$	$-1.11852$	$-1.11803$
1.0	$-1.41421$	$-1.4140$	$-1.41620$	$-1.41421$
5.0	$-2.44949$	$-2.4493$	$-2.44830$	$-2.44949$

**Table 2:** Comparison of $f''(0)$ when $A = 1, M = K = \beta = Pr = R = Bi = 0.0$ for some particular values of *B*.

*B*	Ref.^51^	Ref.^52^	Present results
0.01	$-0.9980$	$-0.9980$	$-0.9980$
0.1	$-0.9694$	$-0.9694$	$-0.9694$
0.2	$-0.9181$	$-0.9181$	$-0.9181$
0.5	$-0.6673$	$-0.6673$	$-0.6673$
2.0	2.0175	2.0175	2.0175
3.0	4.7293	4.7293	4.7293

**Table 3:** Comparison of $\theta ^{'} (0)$ when $A=1, Pr = 10, M = K = \beta = R = B = 0.0$ for various values of *Bi*.

*Bi*	Ref.^53^	Ref.^54^	Present results
0.05	0.0468	0.04679	0.04679
0.10	0.0879	0.08793	0.08794
0.20	0.1569	0.15690	0.15690
0.40	0.2582	0.25818	0.25817
0.60	0.3289	0.32895	0.32895
0.80	0.3812	0.38119	0.38118
1.0	0.4213	0.42134	0.42134
5.0	0.6356	0.63556	0.63556
10.0	0.6787	0.67872	0.67872
20.0	0.7026	0.70256	0.70255

## Discussion on graphical outcomes

Here significance of different control physical parameters of the projected problem on the flow velocity $(f'(\eta ))$, Skin friction $(Re^{\frac{1}{2}}C_{f_x})$, temperature $(\theta (\eta ))$ and heat transfer $(Re^{-\frac{1}{2}}Nu_x )$ are discussed and presented through graphs.

**Figure 2:**
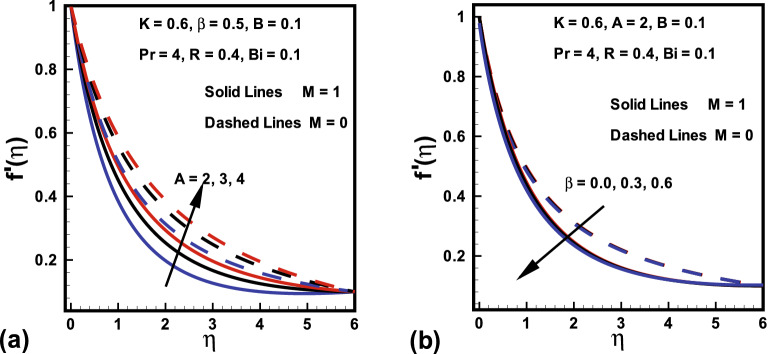
Variations in $f'(\eta )$ (**a**) *A* for $M=0$ and $M=1$ (**b**) $\beta$ for $M=0$ and $M=1$.

**Figure 3:**
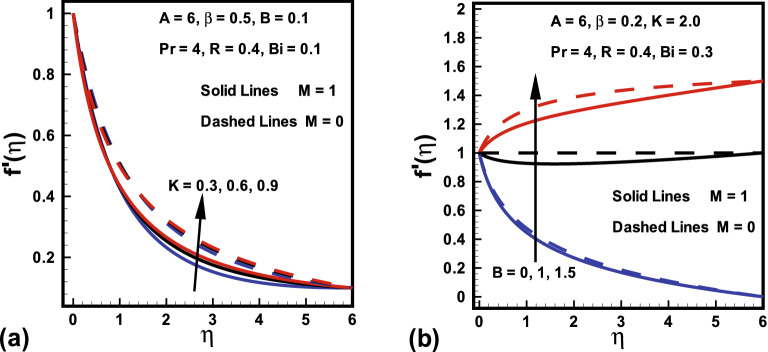
Variations in $f'(\eta )$ (**a**) *K* for $M=0$ and $M=1$ (**b**) *B* for $M=0$ and $M=1$.

**Figure 4:**
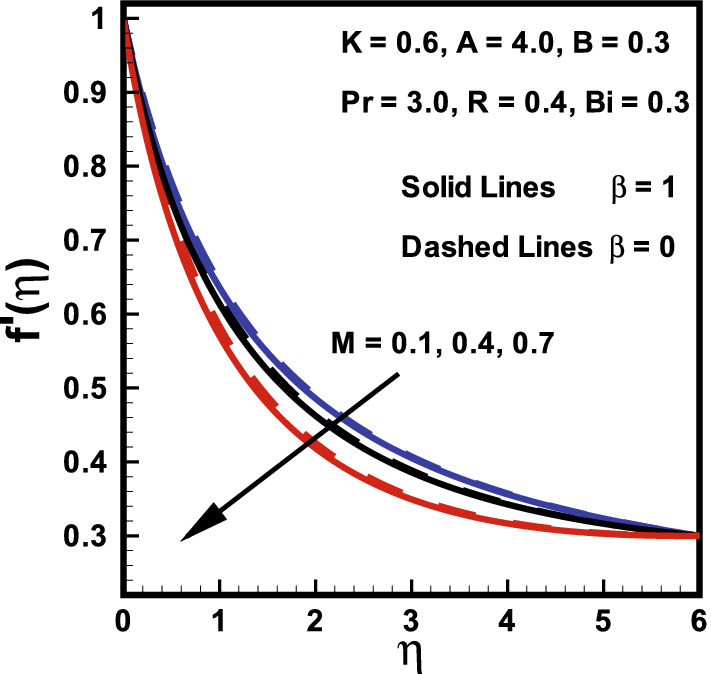
Impact of *M* and $\beta$ on $f'(\eta )$.

Figures 2, 3, 4 demonstrated the influences of distinct values of fluid parameters *A* and $B_1$, magnetic parameter *M*, curvature parameter *K* and ratio of velocities *B* over velocity gradients. Figure 2a portrays the features of fluid parameter *A* on the fluid velocity for both cases ($M=0, \; and \; M=1$), while remaining parameters are kept fixed. It is concluded from this graph that a rise in values of *A* causes boosts up $f'(\eta )$ and momentum boundary layer thickness. Because the higher values of *A* tend to diminish the viscosity and this overcomes the resistance offered to the liquid. Therefore, boundary layer thickness enhances. It is further remarked that $f'(\eta )$ in the absence of *M* shows larger value compared to the velocity field in the presence of *M*. The similar trend was also reported by Hussain et al.^45^. Figure 2b shows that fluid velocity gradient tends to reduce due to rise in fluid parameter $\beta$. It holds physically because $\beta$ varies inversely with momentum diffusivity, which causes a reduction in velocity gradient. Relatively, the $\beta$ variation in presence of *M* shows lesser velocity than the absence of magnetic field. The influence of curvature parameter *K* over dimensionless velocity field in both cases ($M=0, \; and \; M=1$) is presented in Fig. 3a. Here it is revealed from the plot that both the velocity and thickness of the momentum layer rises for *K* in the absence of *M*. In fact *K* varies inversely with radius of cylinder. Thus larger estimation of *K* decays the cylinder radius and hence contact zone of the cylinder with fluid diminishes. Hence less resistive force occurs for the fluid and consequently velocity field improves. Behavior of velocity ratio parameter on the dimensionless fluid velocity in the presence/absence of *M* is sketched in Fig. 3b. Here, $f'(\eta )$ is higher against higher *B* values due to higher free stream velocity. Furthermore, when $U_0$ dominates over $U_\infty$, then $f'(\eta )$ diminishes for larger *B* It is also noted from Fig. 2b that for $B=1$ there is no boundary layer as the free stream and stretching velocities are equivalent. On the other hand, fluid velocity in case of $M=0$ diminishes. Similarly, Fig. 4 is prepared to show the behavior of magnetic parameter *M* with and without fluid parameter $\beta$ while retaining the remaining parameters fixed on the $f'(\eta )$ against $\eta$. It is revealed from Fig. 4 that an increase in the *M* values causes a rise in both the velocity and thickness of momentum layer. It holds physically that a rise in *M* causes an increase in Lorentz force, thus $f'(\eta )$ declines. Moreover, the flow field is more influenced with *M* when $\beta = 1$.

**Figure 5:**
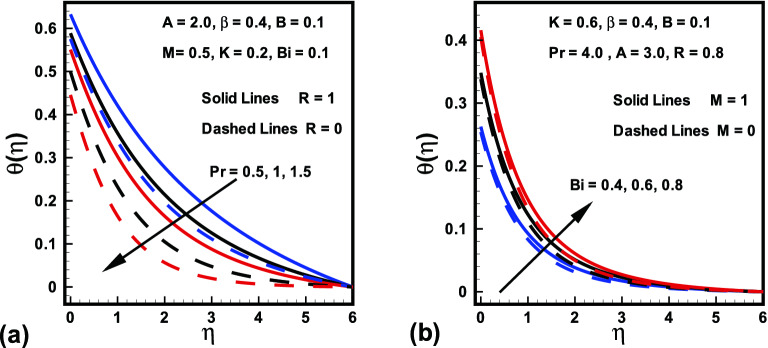
Variations in $\theta (\eta )$ (**a**) *Pr* for $R=0$ and $R=1$ (**b**) *Bi* for $M=0$ and $M=1$.

**Figure 6:**
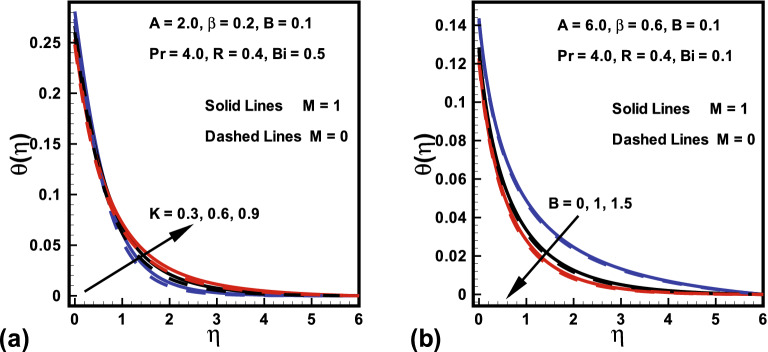
Variations in $\theta (\eta )$ (**a**) *K* for $M=0$ and $M=1$ (**b**) *B* for $M=0$ and $M=1$.

**Figure 7:**
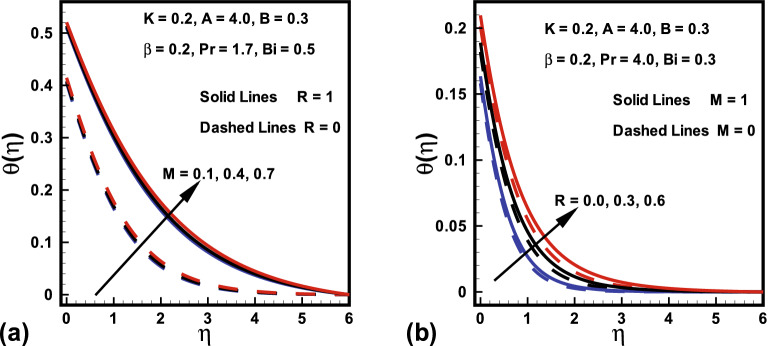
Variations in $\theta (\eta )$ (**a**) *M* for $R=0$ and $R=1$ (**b**) *R* for $M=0$ and $M=1$.

The effects of radiation parameter *R*, magnetic parameter *M*, Prandtl number *Pr*, curvature parameter *K* and Biot number *Bi*, over dimensionless temperature field are plotted in Figs. 5, 6, 7. Figure 5a is designed to show the behavior of Prandtl number *Pr* on the temperature against $\eta$ with and without radiation parameter *R*. It is evident that temperature down with improvement in *Pr*. Because by enhancing *Pr*, the fluid thermal diffusion declines, which accordingly drops the temperature and corresponding thermal layer. Additionally, the temperature field with *R* shows more heat transfer compared to the temperature field without radiation. The significance of Biot number *Bi* over the temperature for both cases ($M=0$ and $M=1$) is displayed in Fig. 5b. It is investigated from the plot that temperature and thickness of the related layer are enhancing functions of *M* and *Bi*. Higher values of *Bi* results in higher heat transfer coefficient which consequently boosts the temperature field. The influence of curvature parameter *K* in the presence/absence of magnetic parameter *M* over dimensionless temperature field is witnessed in Fig. 6a. It is clearly analyzed that for higher *K* near the surface thickness of thermal layer declines whereas it rises far away from the surface with *M*. It holds physically that rise in *K* causes an enhance in heat transfer due to which temperature distribution falls adjacent to the surface, on the other hand, it is the reason for rising the ambient temperature distribution. Figure 6b reveals that fluid temperature declines an increment in the ratio of velocities *B*. However, opposite behavior is found for magnetic parameter *M* on fluid temperature (see Fig. 7a). Because Lorentz force rises for higher *M* and consequently more heat is added which gives rise to temperature field. More improvement is observed when radiation parameter *R* is presented. Similarly, Fig. 7b highlight the behavior of fluid temperature against $\eta$ for radiation parameter *R* in the presence/absence of *M*. It is witnessed from the graph that an increase in *R* causes a boost in the temperature distribution of the flow. This is because a rise in *R* generates the heat energy to the flow, as a result, the thermal layer thicknesses enhances. Also, fluid acquires high temperature in the presence of *M*.

**Figure 8:**
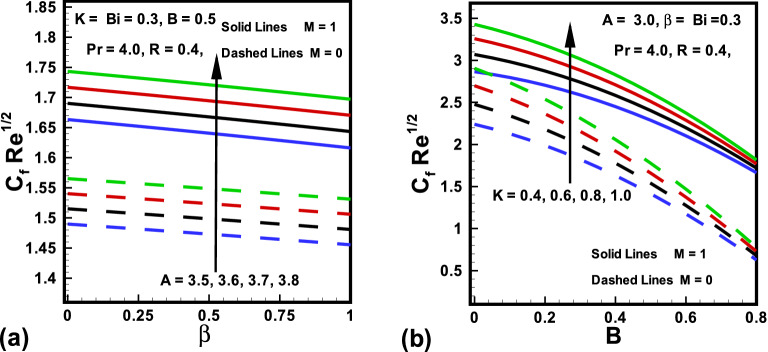
Variations in $Re^{\frac{1}{2}}C_{f_x}$ (**a**) *A* against $\beta$ for $M=0$ and $M=1$ (**b**) *K* against *B* for $M=0$ and $M=1$.

**Figure 9:**
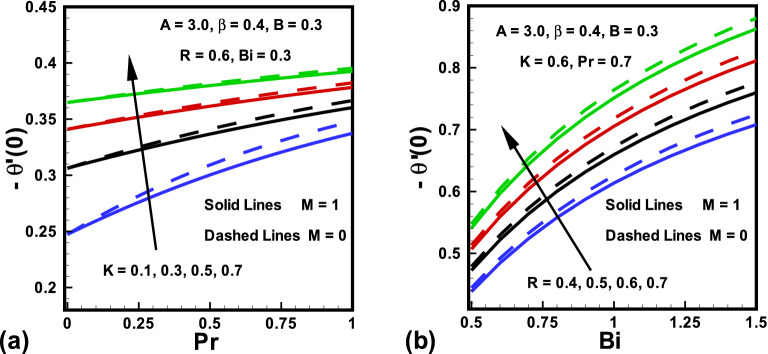
Variations in $Re^{-\frac{1}{2}}Nu_x$ (**a**) *K* against *Pr* for $M=0$ and $M=1$ (**b**) *R* against *Bi* for $M=0$ and $M=1$.

The skin friction coefficient ($Re^{\frac{1}{2}}C_{f_x}$) and Nusselt number $(Re^{-\frac{1}{2}}Nu_x )$ variation due to the change in emerging parameters in the presence/absence of *M* are sketched in Figs. 8 to 9. It is perceived from Fig. 8 that the magnitude of the skin friction rises with magnetic parameter *M*. This is because *M* creates an opposing force which diminishes the fluid velocity and consequently, the skin friction rises for larger values of *M*. The results investigated in Fig. 8a shows that, the fluid parameters *A* and $\beta$ have opposite behavior on the skin friction. Additionally, it is detected from Fig. 8b that as *K* boosts the $Re^{\frac{1}{2}}C_{f_x}$ also boosts. Physically, velocity field at the surface of a cylinder is higher compared to that of a flat plate. On the other hand, the magnitude of the skin friction declines with rising values of *B*. Similarly, the behaviors of curvature parameter *K*, Prandtl number *Pr*, radiation parameter *R* and Biot number *Bi* in the presence/absence *M* on Nusselt number are witnessed in Fig. 9. It is revealed from Fig. 9 that the magnitude of heat transfer is higher in absence of *M*. It is further explained in Fig. 9a that the magnitude of heat transfer is boosted for an increasing values in curvature parameter *K*. It is evidently analyzed that for higher *K* near the surface thickness of thermal boundary layer declines. From this Figure, it is investigated that with rise in *Pr* heat transfer rises. This is because *Pr* declines the fluid temperature which enhances the gap between fluid and surface temperature. Finally, it is revealed from Fig. 9b that the magnitude of heat transfer is higher for larger values of Biot number *Bi* and radiation parameter *R*.

## Conclusion

Here the numerical simulation of a 2D stagnation-point flow of MHD Prandtl–Eyring fluid over a stretching cylinder has been inspected. Further, convective boundary condition and radiation effect are also considered in this study. The computations of converted set of non-linear ODEs are performed successfully by Shooting method numerically using Mathematica software 11. The following are some of the significant findings from the present work:

- It is investigated that fluid velocity decays for higher values of magnetic parameter *M* while the fluid temperature enhances.
- Velocity field improves for fluid parameter *A*, curvature parameter *K* and ratio of velocities *B*; while decreasing function of fluid parameter *B*.
- Further, it is revealed that dimensionless fluid velocity and related layer thickness are enhancing functions of curvature parameter *K*, Biot number *Bi* and radiation parameter *R*; while decreasing functions of Prandtl number *Pr* and ratio of velocities *B*.
- It is concluded that the skin friction boosts by enhancing the fluid parameter *A*, curvature parameter *K* and magnetic parameter *M*.
- The heat transfer rate is boosted for Biot number *Bi*, radiation parameter *R*, Prandtl number *Pr* and curvature parameter *K*.
- Comparative study shows that current outcomes have better relevance with existing results.
